# Effects of fixed versus variable task prioritization during short-term dual task practice on motor and cognitive task performance in young adults

**DOI:** 10.1186/s13104-022-06034-y

**Published:** 2022-05-05

**Authors:** Thomas Muehlbauer, Hagen Voigt, Dennis Brueckner, Rainer Beurskens

**Affiliations:** 1 Division of Movement and Training Sciences/Biomechanics of Sport, University of Duisburg-Essen, Gladbecker Str. 182, 45141 Essen, Germany; 2 Department of Health and Social Affairs, FHM Bielefeld—University of Applied Sciences, Bielefeld, Germany

**Keywords:** Postural control, Skill acquisition, Attention, Resource allocation, Young adults

## Abstract

**Objective:**

It has been shown that variable compared to fixed task prioritization during dual task practice more effectively improves motor (i.e., postural control) and cognitive (i.e., memory) performance in older adults. However, it is unclear whether this finding is also valid in young adults. Thus, the present study examined the effect of fixed (allocate equal priority on both tasks) versus variable (vary priority between both tasks) priority during short-term motor-cognitive dual task practice on single and dual task performance in healthy young adults (age range: 20–30 years).

**Results:**

During two days of practice, significant improvements of motor (i.e., balance task: reduced root mean square error; *p* < 001, *η*_p_^2^ = .72) and cognitive (i.e., arithmetic task: increased serial three subtractions; *p* < .001, *η*_p_^2^ = .78) task performance were observed and that was irrespective of group (“fixed priority” and “variable priority”). Further, the statistical analysis of post-practice single and dual task performance revealed no significant differences between groups, irrespective of task (i.e., motor or cognitive). This indicates that in young as opposed to old adults, single and dual task performance improvements are independent of task prioritization (i.e., fixed or variable priority) during short-term motor-cognitive dual task practice.

## Introduction

Motor-cognitive dual task (DT) situations regularly occur in activities such as climbing stairs while talking on the cell phone and require an effective allocation of limited attentional resources to be mastered successfully. In order to meet this requirement, motor-cognitive DT practice programs have been developed and their effectiveness has been demonstrated in numerous original studies [1–3] and systematic reviews [4, 5]. Contrary, few investigations addressing the efficient design of exercise modalities used for motor-cognitive DT practice exist. For example, it remains open how to best allocate attention when practicing two tasks simultaneously. Attention can either be equally allocated to both tasks during DT practice or prioritized to one of the two tasks. In this regard, Beurskens et al. [6] were able to show that DT performance improved, irrespective of task prioritization during DT practice (i.e., fixed prioritization of the motor or cognitive task). Besides the aforementioned fixed task prioritization, it is possible to vary task prioritization during motor-cognitive DT practice. Specifically, first the motor and then the cognitive task or vice versa could be prioritized during practice. Several studies [7–9] have shown that this results in a different training effectiveness. Silsupadol et al. [7] showed DT training (4 weeks, 3 times/week) with variable compared to fixed task prioritization was more effective in improving both motor (i.e., postural control) and cognitive (i.e., memory) DT performance in individuals aged > 65 years. Despite this gain in knowledge, this study [7] and further studies [8, 9] are limited to older adults. Therefore, it remains unclear whether prioritization effects during short-term motor-cognitive DT practice [6, 10] also occur in young adults who, compared to seniors, also showed performance declines in DT situations in at least one of the given tasks [11].

Thus, the study aim was to investigate the effects of fixed compared to variable priority instructions during short-term motor-cognitive DT practice on single task (ST) and DT performance in healthy young adults. A variable allocation of attentional resources during DT practice requires switching of task prioritization and shifting attention involves a stronger involvement of neural processing mechanisms [12]. We hypothesized that both practice conditions lead to improvements, but superior effects are expected during variable compared to fixed priority motor-cognitive DT practice.

## Main text

### Methods

#### Participants

An a priori power analysis using G*Power [13] with the following input parameters was performed: *f* = 0.25, *α* = 0.05, 1−*β* = 0.80, groups (*n* = 2), measurements (*n* = 2), correlation between measurements (*r* = 0.6) and revealed a total sample size of *N* = 28 participants. Twenty-eight young adults were recruited and randomly assigned (by coin flip) to either a “fixed priority” (*n* = 14; age: 26.4 ± 2.4 years, 7 females and 7 males) or a “variable priority” (*n* = 14; age: 25.6 ± 2.6 years, 7 females and 7 males) motor-cognitive DT practice group.

#### Experimental procedures

**Fig. 1:**
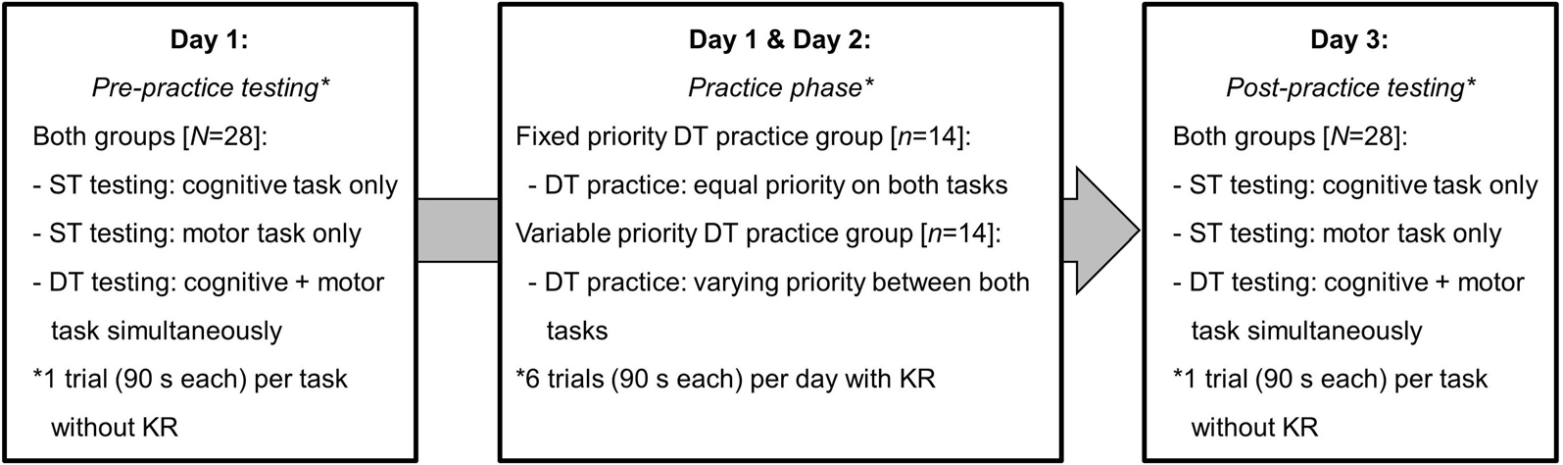
Schematic description of the experimental study design. *DT*   dual task, *KR*  knowledge of result, *ST* single task

The design of the study is displayed in Fig. 1. On day 1, all participants performed a pre-practice testing sequence for one 90-s trial per task: (a) cognitive task only, (b) motor task only, (c) cognitive and motor task simultaneously. Immediately after this as well as on day 2, all participants were asked to perform the cognitive and the motor task simultaneously for six 90-s trials each day. Participants of the “fixed priority” group were instructed to allocate equal priority on both tasks. Subjects of the “variable priority” group were asked to vary priority between the tasks. Specifically, the motor task was to be prioritized in trials 1–3 and the cognitive task in trials 4–6. During practice, both groups received feedback (i.e., total number of correct responses and time in balance) after each trial and the inter-trial rest was 90 s. On day 3, all participants conducted the post-practice testing using the same testing sequence as during pre-practice testing.

#### Tasks

The motor task was a balancing task. Participants were instructed to balance on an unstable wooden stability platform (Lafayette Instrument, USA) and to keep the platform horizontal [1]. A timer measured time in balance, which was defined as the time when the platform was within ± 3° of the horizontal position at a rate of 25 Hz. Platform position data were exported and used to calculate the root-mean-square error (RMSE). The lower the RMSE, the better the motor performance.

The cognitive task was an arithmetic task. Participants were asked to loudly recite serial three subtractions starting from a randomly selected number from 300–900 that was provided by the experimenter [2]. In case of miscalculation, the false calculation was noted. When correctly continuing the subtractions, only one error was noted (i.e., no consequential errors were registered). The number of correct subtractions (i.e., total number of subtractions minus the number of subtraction mistakes) was used as outcome measure. The higher the total number of correct subtractions, the better the cognitive performance.

#### Statistical analysis

Descriptive statistics were presented as means ± SD. Normal distribution was examined using the Shapiro–Wilk test (*p* > 0.05) and homogeneity of variances using the Levene test (*p* > 0.05). A 2 (Group: “fixed priority”, “variable priority”) × 2 (Task: single, dual) ANOVA with repeated measures on Task was used to detect task-dependent group differences during pre- and post-practice testing. Further, a 2 (Group: “fixed priority”, “variable priority”) × 2 (Day: 1–2) × 6 (Trial: 1–6) ANOVA with repeated measures on Day and Trial was performed to assess group discrepancies during the practice phase. Partial eta-squared was calculated and reported as small (0.02 ≤ *η*_p_^2^ ≤ 0.12), medium (0.13 ≤ *η*_p_^2^ ≤ 0.25), and large (*η*_p_^2^ ≥ 0.26). All analyses were performed using the SPSS and significance levels were set at *p* < 0.05.

## Results

**Fig. 2:**
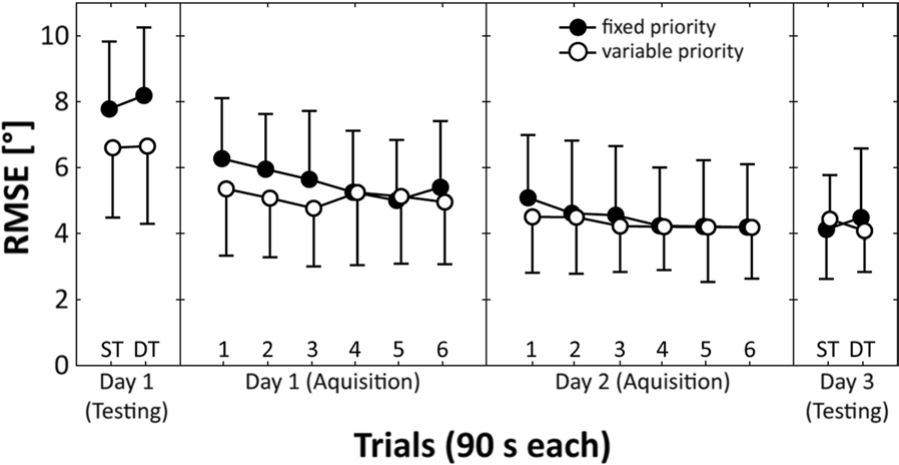
Root mean square error (RMSE) during pre-practice testing (Day 1), practice phase (Day 1 and Day 2), and post-practice testing (Day 3) for the “fixed priority” (filled circles) compared to the “variable priority” (unfilled circles) practice group. *DT* dual task, *ST* single task

**Fig. 3:**
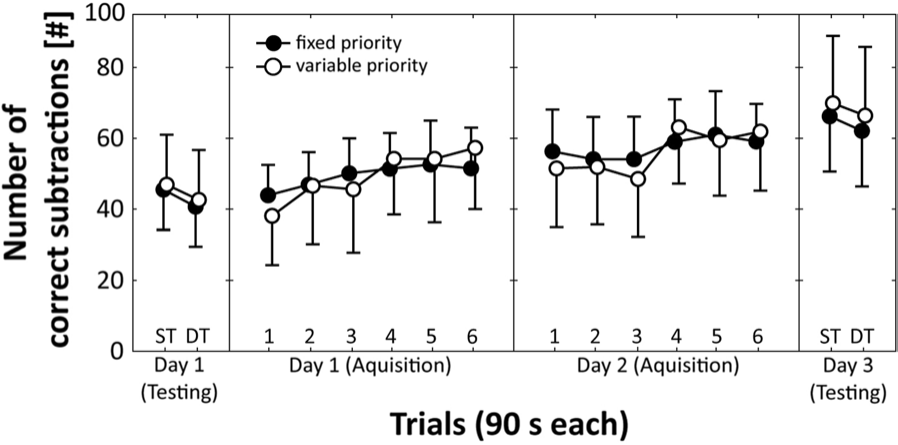
Total number of correct subtractions during pre-practice testing (Day 1), practice phase (Day 1 and Day 2), and post-practice testing (Day 3) for the “fixed priority” (filled circles) compared to the “variable priority” (unfilled circles) practice group. *DT*  dual task, *ST*  single task

Motor and cognitive task performance are displayed in Fig. 2, 3, respectively.

### Pre-practice testing (day 1)

The main effect of Group (motor task: *F*_(1,26)_ = 3.366, *p* = 0.078, *η*_p_^2^ = 0.12; cognitive task: *F*_(1,26)_ = 0.139, *p* = 0.712, *η*_p_^2^ = 0.01) and the Group × Task interaction were not significant (motor task: *F*_(1,26)_ = 0.278, *p* = 0.602, *η*_p_^2^ = 0.01; cognitive task: *F*_(1,26)_ = 0.022, *p* = 0.884, *η*_p_^2^ = 0.01). Further, the main effect of Task was significant for the cognitive (*F*_(1,26)_ = 7.135, *p* = 0.013, *η*_p_^2^ = 0.22) but not for the motor task (*F*_(1,26)_ = 0.490, *p* = 0.490, *η*_p_^2^ = 0.02), indicating that irrespective of group the number of correct subtractions was lower during DT compared to ST testing.

### Practice phase (day 1 and 2)

For both measures, ANOVA yielded significant main effects of Day (motor task: *F*_(1,26)_ = 65.456, *p* < 0.001, *η*_p_^2^ = 0.72; cognitive task: *F*_(1,26)_ = 91.491, *p* < 0.001, *η*_p_^2^ = 0.78) and Trial (motor task: *F*_(5,135)_ = 11.115, *p* < 0.001, *η*_p_^2^ = 0.30; cognitive task: *F*_(5,135)_ = 48.850, *p* < 0.001, *η*_p_^2^ = 0.64) but not of Group (motor task: *F*_(1,26)_ = 0.272, *p* = 0.606, *η*_p_^2^ = 0.01; cognitive task: *F*_(1,26)_ = 0.016, *p* = 0.899, *η*_p_^2^ = 0.01), indicating performance enhancements across days and trials. The Group × Day × Trial interactions were not significant (motor task: *F*_(5,130)_ = 0.963, *p* = 0.443, *η*_p_^2^ = 0.04; cognitive task: *F*_(5,130)_ = 0.665, *p* = 0.651, *η*_p_^2^ = 0.03), indicating that improvements did not depend on the practiced priority condition.

### Post-practice testing (day 3)

Again, the main effect of Group (motor task: *F*_(1,26)_ = 0.008, *p* = 0.929, *η*_p_^2^ = 0.01; cognitive task: *F*_(1,26)_ = 0.382, *p* = 0.542, *η*_p_^2^ = 0.01) and the Group × Task interaction were not significant (motor task: *F*_(1,26)_ = 3.199, *p* = 0.085, *η*_p_^2^ = 0.11; cognitive task: *F*_(1,26)_ = 0.041, *p* = 0.842, *η*_p_^2^ = 0.01). In addition, the main effect of Task was significant for the cognitive (*F*_(1,26)_ = 7.134, *p* = 0.013, *η*_p_^2^ = 0.22) but not for the motor task (*F*_(1,26)_ = 0.001, *p* = 1.0, *η*_p_^2^ = 0.01), indicating that the number of correct subtractions was lower during DT compared to ST testing, irrespective of prioritization.

## Discussion

Contrary to our hypothesis, no significant group differences during post-practice testing neither for the cognitive nor for the motor task under ST and DT conditions were found. Thus, short-term motor-cognitive DT practice under fixed compared to variable priority instructions did not result in group-specific learning improvements. Our finding contradicts previous work. Silsupadol et al. [7] randomly assigned older adults to one of three groups: i) DT balance training group with fixed task prioritization, ii) DT balance training group with variable task prioritization, iii) ST balance training group. After four weeks of training, DT training with variable priority instructions was more effective in improving motor (i.e., gait behavior) and cognitive (i.e., response rate) DT performance than DT training with fixed priority instructions or ST training. Further, Silsupadol et al. [8] investigated motor and cognitive DT performance following four weeks of DT balance training with fixed or variable task prioritization or ST balance training in seniors. Authors detected larger motor (i.e., gait speed) and cognitive (i.e., successful trial number) task improvements during DT testing for participants who received DT training with variable priority instructions compared to those with fixed priority instructions and those performing ST training. Lastly, Lussier et al. [9] examined older adults conducting five one-hour sessions of training with fixed or variable priority instructions or an active placebo (i.e., computer classes). Following intervention, significantly larger improvements in a near and far modality transfer task for participants that trained with variable task prioritization were observed.

The better performances under variable versus fixed task prioritization during DT task practice is justified, for example, by the fact that the former versus the latter requires a shifting of attention between two tasks and thus a greater involvement of neural processing [12]. Specifically, Zendel et al. [12] observed an increase in peak amplitude of the N200 wave using EEG recordings that was associated with enhanced DT performance (i.e., alphanumeric equation plus visual detection task) in older adults performing variable divided attention training in six one-hour sessions compared to those with fixed divided attention training or ST training. Moreover, particularly during variable priority DT practice it is emphasized that there is a relationship of two different tasks (i.e., motor vs. cognitive) [14]. Finally, the change in task prioritization creates variation during variable priority DT practice and thus contextual interference, which according to Shea and Morgan [15] has a positive effect on learning processes. However, in the present study this does not seem to result in better post-practice performance in the “variable priority” group. Beside methodological differences (i.e., application of different motor [e.g., stabilometer vs. obstacle crossing] and cognitive tasks [e.g., serial three subtractions vs. auditory Stroop task] in the present compared to former studies), a possible reason could be age-differences of participants. The present study examined young adults while in all previous studies, older adults were examined. Therefore, it can be assumed that aging processes have a modulating impact on the effectiveness of different priority instructions during motor-cognitive DT and thus the design of task prioritization becomes significant only at older ages. Further, the applied practice period of two days [6, 10] was much shorter compared to previous studies where training periods ranged from five hours to four weeks [7–9]. Future studies should therefore examine whether longer practice duration and thus stronger adaptation processes lead to differences between fixed compared to variable priority instructions during motor-cognitive DT practice in young adults.

## Conclusion

Short-term motor-cognitive DT practice effectively improved motor (i.e., reduced RMSE) and cognitive (i.e., increased total number of correct subtractions) performance under ST and DT testing in young adults, irrespective of task prioritization (fixed or variable priority). Thus, the type of attentional resource allocation during DT practice does not play a major role for the effectiveness of a DT practice regime in young adults. This is contrary to studies with older adults and indicates that only with increasing age the importance of task prioritization during DT practice becomes significant.

## Limitations

- The lack of a control group limits the validity of the observed pre- to post-practice changes.
- The observed effects only apply to short-term (i.e., several days) but not to longer term (i.e., several weeks or months) practice periods.

## Data Availability

The data generated and analyzed during the present study are available from the corresponding author upon reasonable request.

## Abbreviations

ANOVA: Analysis of variance
DT: Dual task
RMSE: Root mean square error
SD: Standard deviation
SPSS: Statistical package for social sciences
ST: Single task
